# Editorial: Application of metabolomics, peptidomics and proteomics in human nutrition and health

**DOI:** 10.3389/fchem.2024.1504501

**Published:** 2024-10-16

**Authors:** Evroula Hapeshi, Victoria Samanidou, Ioannis Sarigiannis, Christos Petrou, Panagiotis Vorkas

**Affiliations:** ^1^ Department of Health Sciences, School of Life and Health Sciences, University of Nicosia, Nicosia, Cyprus; ^2^ Bioactive Molecules Research Center (BioMoReC), University of Nicosia, Nicosia, Cyprus; ^3^ Laboratory of Analytical Chemistry, School of Chemistry, Aristotle University of Thessaloniki, Thessaloniki, Greece; ^4^ Department of Surgery and Cancer, Faculty of Medicine, Imperial College London, South Kensington Campus, London, United Kingdom

**Keywords:** proteomics, peptidomics, metabolomics, mass spectrometry, human health, analytical techniques, metabolomic pathway, food digestion

Molecular profiling strategies assist in selecting treatments and improving patient management by identifying disease biomarkers and classifying patients into biological subgroups. Proteomics and peptidomics are valuable for clinical assessment, aiding in prognosis, diagnosis, and treatment response, while also facilitating the discovery of effective therapies and novel therapeutic targets. Recently, peptidomics and proteomics have expanded into food and nutrition research, identifying peptides in food products and during processing. Metabolomics, similar to proteomics and peptidomics, evaluates metabolic pathways in clinical and food samples, benefiting from advancements in mass spectrometry and NMR for detailed profiling.

This Research Topic focuses on developing and applying proteomics, peptidomics, and metabolomics in clinical and food science to understand proteins, peptides, and metabolites, and their health impacts. It explores trends in these fields for discovering disease biomarkers, drug development, and identifying bioactive peptides and food digestion biomarkers.

Although numerous studies have focused on transcriptome analysis, shell protein characteristics, and intestinal flora in *Viviparidae* snails, research in the areas of proteomics and phosphoproteomics remains limited. According to Liu et al., examining the proteomic and phosphoproteomic profiles of *C. chinensis* from various geographical regions and environmental conditions provides valuable insights into metabolic pathways and biological functions. This research may also uncover new nutritional and medicinal components in *C. chinensis*. Individual *C. chinensis* exhibit variations depending on their location and environment. Several novel proteins have been identified in *C. chinensis* from China. The proteomic analysis revealed a total of 1,382 proteins, with 690 being quantified. Additionally, 1,039 phosphorylated proteins were identified and quantified. The analysis indicated that differentially expressed proteins (DEPs) are crucial in metabolic processes, localization, and biological regulation. Phosphorylated proteins may participate in signaling pathways and protease regulation, including guanylate cyclase, tyrosine protein kinase, receptor protein tyrosine kinase, and glyoxylate reductase/hydroxypyruvate reductase. Consequently, these proteomic profiles enhance our understanding of food nutrition, signaling pathways, and metabolic mechanisms in *C. chinensis* (Liu et al.).

Recent advancements in metabolomics have led to the development of biomarkers of food intake (BFIs) and the study of diet-microbiome interactions. While candidate BFIs for meat and dairy intake exist, further validation is needed. BFIs are promising for objective dietary assessment, potentially linking diet to health outcomes. Using ultra-high performance liquid chromatography coupled with high-resolution mass spectrometry (UHPLC-HRMS), 38 biomarkers for meat and dairy intake have been identified. These biomarkers were selected, identified, and confirmed through rigorous methods and categorized into four elimination kinetics trajectories to optimize urine sampling times for dietary studies (La Barbera et al.). Additionally, metabolites from gut microbial proteolysis, which can affect health, have been studied. Differences in microbial metabolism after dairy and meat meals have been assessed, with certain metabolites indicating potential as BFIs for dairy. Some metabolites, like 3-indoxyl sulfate, suggest a dual origin from both dairy and gut metabolism, necessitating their inclusion in combined BFI panels. Further research is needed to determine the sources of these metabolites in urine after protein-rich meals (La Barbera et al.).

Most applied proteomics studies are conducted with heterogeneous cell populations, leading to a loss of protein content information specific to individual cells. Analyzing cells individually can eliminate this dilution effect, but single-cell proteomic analysis is challenging due to the low protein levels available for sample preparation and mass spectrometric analysis. A robust, Thermal inkjet (TIJ)-enabled, label-free single-cell proteomic workflow has been introduced, which is accessible to the research community due to its affordability and reliance on commonly available components (Stanisheuski et al.). This method has successfully achieved the label-free identification of up to 1,300 proteins from a single cell in a single run. The protocol’s development and applicability for proteomics of single cells from various cell lines, mixed cell suspensions, and glioblastoma tumor spheroids have been demonstrated. This cost-effective and reliable single-cell proteomics workflow can be adopted by other laboratories interested in studying cells at the individual level (Stanisheuski et al.).

In recent years, peptidomic strategies have been used to study modifications of antimicrobial peptides (AMPs) to enhance their activity. N-capping and C-capping, involving specific amino acids or unconventional motifs, can alter peptide secondary structure and improve activity against pharmacological targets. These capping motifs help prevent peptide degradation and optimize peptide variants. Brango-Vanegas et al. discuss strategies for creating N- and C-cap motifs to refine AMPs. Understanding these effects could allow customization of AMPs for specific infections or drug delivery, addressing the need for new anti-infectives amid rising antimicrobial resistance. Capping motifs are promising for developing next-generation AMP therapeutics with better efficacy and safety (Brango-Vanegas et al.).

Recently, the scientific community has focused on applying peptidomics, proteomics, and metabolomics in clinical contexts, such as discovering disease biomarkers and developing drugs. The carcinogenic effects of environmental chemicals are linked to their metabolic activation and DNA damage. In breast cancer, the potency of these compounds is influenced by their composition and the overexpression of AhR and SULT1A1 proteins, with SULT1A1 as a potential biomarker for targeted therapy. Baker et al. explored the role of the Aryl hydrocarbon Receptor (AhR) in breast cancer and the development of therapeutic molecules. The study showed that CYP1 metabolism occurs at the phenyl head group, with certain substituents reducing cytotoxicity. Further research examined the selectivity and activation of the AhR/CYP1/SULT1A1 axis in breast cancer using cell line models (Baker et al.).

The results clearly indicate that applying peptidomics, proteomics, and metabolomics in clinical and food science is essential for evaluating and understanding proteins, peptides, and metabolites in clinical samples and food digestion, as well as their impact on human health. In the future, more selective and sensitive strategies in peptidomics, proteomics, and metabolomics will be developed and applied for discovering disease biomarkers, drug development, and identifying novel therapeutic targets. Additionally, these strategies will be used in food research to identify bioactive peptides and biomarkers related to food digestion.

